# Sleep Quality Improvement Enhances Neuropsychological Recovery and Reduces Blood Aβ_42/40_ Ratio in Patients with Mild–Moderate Cognitive Impairment

**DOI:** 10.3390/medicina57121366

**Published:** 2021-12-15

**Authors:** Haihua Huang, Mingqiu Li, Menglin Zhang, Jiang Qiu, Haiyan Cheng, Xin Mou, Qinghong Chen, Tina Li, Jun Peng, Benyi Li

**Affiliations:** 1 Department of Geriatrics, Jianghan Oilfield General Hospital, The Yangtze University School of Medicine, Qianjiang 433121, China; lmqaiyy@yangtzeu.edu.cn (M.L.); qjmouxing@yangtzeu.edu.cn (X.M.); qjqinghong@yangtzeu.edu.cn (Q.C.); 2 Hubei Clinical Research Center of Dementia and Cognitive Impairment, Wuhan 434300, China; 3 Health Science Center, The Yangtze University School of Medicine, Jingzhou 434023, China; 201972562@yangtzeu.edu.cn; 4 Department of Clinical Laboratory, Jianghan Oilfield General Hospital, The Yangtze University School of Medicine, Qianjiang 433121, China; teddy02514028@yangtzeu.edu.cn; 5 Division of Research & Education Administration, Jianghan Oilfield General Hospital, The Yangtze University School of Medicine, Qianjiang 433121, China; chytcm@yangtzeu.edu.cn; 6 Department of Urology, The University of Kansas Medical Center, Kansas City, KS 66160, USA; tina.Li@rice.edu; 7 Department of Nursing, Jianghan Oilfield General Hospital, The Yangtze University School of Medicine, Qianjiang 433121, China; pjhl1974@yangtzeu.edu.cn

**Keywords:** mild–moderate cognition impairment, sleep-disordered breathing, Tau-pT181, neuropsychologic symptom, depression, anxiety

## Abstract

*Background and objectives*: Alzheimer’s disease is a progressive brain degeneration and is associated with a high prevalence of sleep disorders. Amyloid β peptide-42/40 (Aβ_42/40_) and Tau-pT181 are the core biomarkers in cerebrospinal fluid and blood. Accumulated data from studies in mouse models and humans demonstrated an aberrant elevation of these biomarkers due to sleep disturbance, especially sleep-disordered breathing (SDB). However, it is not clear if sleep quality improvement reduces the blood levels of Ab_42/40_ ratio and Tau-pT181 in Alzheimer’s disease patients. *Materials and Methods*: In this prospective study, a longitudinal analysis was conducted on 64 patients with mild–moderate cognition impairment (MCI) due to Alzheimer’s disease accompanied by SDB. Another 33 MCI cases without sleep-disordered breathing were included as the control group. Sleep quality was assessed using the Pittsburgh Sleep Quality Index (PSQI) score system. Neuropsychological assessments were conducted using the Montreal Cognitive Assessment (MoCA), Geriatric Depression Scale (GDS), Clinical Dementia Rating (CDR), 24-h Hamilton Rating Scale for Depression (HRSD-24), and Hamilton Anxiety Rating Scale (HAMA) scoring systems. Aβ_42_, Aβ_40,_ and Tau-pT181 protein levels in blood specimens were measured using ELISA assays. All patients received donepezil treatment for Alzheimer’s disease. SDB was managed with continuous pressure ventilation. *Results*: A significant correlation was found among PSQI, HRSD-24, HAMA, Aβ_42/40_ ratio, and Tau-pT181 level in all cases. In addition, a very strong and negative correlation was discovered between education level and dementia onset age. Compared to patients without SDB (33 non-SD cases), patients with SDB (64 SD cases) showed a significantly lower HRSD-24 score and a higher Aβ_42/40_ ratio Tau-pT181 level. Sleep treatment for patients with SDB significantly improved all neuropsychological scores, Aβ_42/40_ ratio, and Tau-pT181 levels. However, 11 patients did not completely recover from a sleep disorder (PSQI > 5 post-treatment). In this subgroup of patients, although HAMA score and Tau-pT181 levels were significantly reduced, MoCA and HRSD-24 scores, as well as Aβ_42/40_ ratio, were not significantly improved. ROC analysis found that the blood Aβ_42/40_ ratio held the highest significance in predicting sleep disorder occurrence. *Conclusions*: This is the first clinical study on sleep quality improvement in Alzheimer’s disease patients. Sleep quality score was associated with patient depression and anxiety scores, as well as Aβ_42/40_ ratio and Tau-pT181 levels. A complete recovery is critical for fully improving all neuropsychological assessments, Aβ_42/40_ ratio, and Tau-pT181 levels. Blood Aβ_42/40_ ratio is a feasible prognostic factor for predicting sleep quality.

## 1. Introduction

Alzheimer’s disease (AD) is a progressive neuronal degenerative disorder, and nearly 50 million people live with dementia worldwide—75% are Alzheimer’s disease patients [1]. AD is the sixth leading cause of death in the US, accounting for more than 122 K deaths in 2018, more than breast cancer and prostate cancer combined [2]. It is estimated that 5.8 million Americans age 65 and older have Alzheimer’s disease this year, and the number is projected to be 13.8 million by 2050, according to the Alzheimer’s Association (www.alz.org accessed on 29 November 2021). Therefore, there is a desperate need for medical breakthroughs to prevent, slow, or cure Alzheimer’s disease.

Alzheimer’s disease has two pathophysiological hallmarks in the brain: interstitial deposition of insoluble amyloid-β (Aβ) peptides and intracellular aggregation of hyperphosphorylated Tau proteins [3]. Aβ deposition and Tau protein aggregation are long-term and slow processes, starting 20 years before any noticeable symptoms, a so-called prodromal phase [4,5]. Aβ peptides are physiologically processed from their precursor protein, mainly in neuronal cells after sequential proteolytic cleavage by β-secretase and γ-secretase [5,6]. There are four significant isoforms of Aβ peptides (38, 40, 42, and 43), which are detectable in brain interstitial fluid (ISF), cerebrospinal fluid (CSF), and blood plasma [7]. Aβ_40_ and Aβ_42_ peptides are more abundant than others. The Aβ_42_ peptide is less soluble due to two extra hydrophobic amino acid residues at the C-terminus, rendering it more prone to deposition than Aβ_40_ [8]. Currently, the levels of Aβ_42/40_ ratio in patient CSF and blood are implicated as disease biomarkers in the clinic [7,9]. Tau protein is a crucial component of microtubule assembly in axons, and its function is regulated by phosphorylation on multiple residues, including threonine 181 (Tau-pT181) [10]. Like Aβ peptides, Tau protein and its pT181 form are also detectable in CSF and blood specimens; their levels are associated with disease progression [11].

In recent years, sleep disorders have been linked to Alzheimer’s disease progression in addition to the typical symptoms of progressive loss of memory, speech, and cognition [12,13]. A high prevalence (25–66%) of Alzheimer’s disease patients was reported to exhibit various sleep disorders, including sleep-disordered breathing (SDB) [14,15]. In contrast, the incidence of sleep disorders was only 18.3–27.6% in elderly adults without cognition impairment [14]. These sleep behavior changes began in the prodromal phase of Alzheimer’s disease, while patients only suffered from mild cognitive impairment (MCI), possibly due to amyloid/Tau pathology before cognition decline [14]. Recent studies showed that more than half of the AD patients also are suffering from SDB [16]. Sleep deprivation or disturbances increased Aβ peptides and Tau proteins in cerebrospinal fluid (CSF) compared to normal sleep controls [9,17,18,19] due to high production of Aβ peptides [20], which was supported by studies from transgenic mouse models of Alzheimer’s disease [21].

Interestingly, sleep treatment with continuous positive airway pressure in SDB patients significantly improved cognition scores in patients with Alzheimer’s disease [22]. On the other hand, a healthy sleep cycle was shown to facilitate Aβ clearance from the brain tissue [23]. These studies suggest a bidirectional relationship between sleep disorders and AD progression [14,24]. However, it is unknown if sleep quality improvement would reduce or slow cognition impairment in Alzheimer’s disease patients.

The purpose of this study was to examine the effect of sleep quality improvement on neuropsychological symptoms and blood levels of Aβ peptides and Tau proteins in Alzheimer’s disease patients with mild–moderate cognition impairments. We were also interested in identifying potential risk factors that provide prognostic feasibility for sleep quality prediction in Alzheimer’s disease patients. We compared the sleep quality (PSQI) score with a longitudinal approach, neuropsychological parameters (Montreal Cognitive Assessment (MoCA), Geriatric Depression Scale (GDS), Clinical Dementia Rating (CDR), 24-h Hamilton Rating Scale for Depression (HRSD-24) and Hamilton Anxiety Rating Scale (HAMA)), blood Aβ_42/40_ ratio, and Tau-pT181 proteins before and after a 6-month sleep treatment in 64 MCI patients with sleep-disordered breathing. Our results revealed that PSQI scores significantly correlated with HRSD-24 and HAMA scores, as well as Aβ_42/42_ ratio and Tau-pT181 levels, but not with MoCA, GDS, or CDR scores. A significant improvement was achieved after sleep treatment for PSQI, MoCA, HRSD-24, HAMA scores, and Aβ_42/40_ ratio and Tau-pT181 levels. Most interestingly, a complete recovery of sleep quality improved neuropsychological scores, Aβ_42/40_ ratio, and Tau-pT181 levels. However, MoCA and HRSD-24 scores, plus Aβ_42/40_ ratio, were not significantly enhanced in the patient who did not completely recover (PSQI > 5 post-treatment). ROC analysis identified Aβ_42/40_ ratio as the most potent predicting factor for sleep disorder occurrence. Our study demonstrated for the first time, as the authors are aware, that sleep quality improvement can enhance the neuropsychological status and reduce blood Aβ_42/40_ ratio and Tau-pT181 levels in MCI patients due to Alzheimer’s disease.

## 2. Materials and Methods

### 2.1. Study Design and Patients

A prospective longitudinal study was designed to examine the effect of sleep quality improvement on neuropsychological behaviors and the changes of blood Aβ_42/40_ ratio and Tau-pT181 levels in patients with mild cognitive impairment due to Alzheimer’s disease accompanied with sleep-disordered breathing. Patients were recruited at the Memory and Sleep Clinic at the Jianghan Oilfield General Hospital from February 2017 to December 2019. The diagnosis of AD dementia was made based on the core diagnostic criteria developed by the National Institute on Aging-Alzheimer’s Association in 2011 [25]. The diagnostic criteria for sleep-disordered breathing in AD patients were based on the definition of “Dementia-related sleep disorders” in the International Classification of Sleep Disorders guidelines [26] and the clinical diagnostic criteria for Alzheimer’s disease-related sleep disorders [27]. Other inclusion criteria include no severe dysfunctions or lesions of the heart, lung, liver, kidney, and other vital organs and the ability to complete relevant neuropsychological assessment and auxiliary examination. Exclusion criteria included: (1) severe dementia; (2) other types of cognitive impairment, including vascular dementia, Parkinson’s disease, frontotemporal dementia, Lewy body dementia; (3) a history of severe mental illness; (4) association with a severely debilitating illness, infectious disease, painful condition or other diseases that may affect the quality of sleep, such as chronic obstructive pulmonary disease, stroke, heart failure, kidney failure, severe cerebrovascular disease, epilepsy; and (5) severe physical movement disorder.

#### 2.1.1. General Procedures

The study protocol was reviewed and approved by the Ethics Committee of the Jiangshan Oilfield General Hospital (study ethical code number 2017016, approval date 20170226). All the patients and their immediate family members were informed with a written consent form, and the patient’s signatures were obtained before enrollment. This study was conducted according to the principles stated in the Declaration of Helsinki [28].

After diagnosis and recruitment, all patients were managed by dedicated research nurses responsible for collecting demographic data, medical history, physical examination, behavioral and neuropsychological assessment, biochemical specimen collection, and regular follow-up. Structured questionnaires were used to assess patient medical history, gender, age, onset age of the disease, course of the disease, years of education, marital status, the living situation at home, and patient support level on the enrollment day. Patients were requested to spend one night at the hospital, where peripheral blood specimens were collected in the morning before daytime activity.

#### 2.1.2. Sleep Quality Evaluation

The Pittsburgh Sleep Quality Index (PSQI) questionnaire (a Chinese version) was used to evaluate patients’ overall sleep quality [29,30]. There were 18 items on the scale divided into seven sub-items: subjective sleep quality, time to sleep, sleep time, sleep efficiency, night sleep disturbance, sleep drug use, and daytime dysfunction. Each item’s score is 0–3 points, and the total score is 0–21 points. A PSQI score at or above five was set as the cutoff value for a sleep disorder.

#### 2.1.3. Assessment of Neuropsychological Status

The Montreal Cognitive Assessment Scale (MoCA) was used to assess patient cognitive function [31]. There were seven cognitive domains: visuospatial and executive function, naming, delayed recall, attention, language, abstraction, and orientation. A total score of less than 26 was considered as cognitive impairment.

The Clinical Dementia Assessment Scale (CDR) was used to provide a global evaluation of the severity of dementia [32]. A CDR score of 1 was classified as mild, 2 as moderate, and 3 as severe. The Global Deterioration Scale (GDS) was used to assess the extent and progress of dementia [33], which provides an overview of a patient who has degenerative dementia. The GDS scale was divided into seven levels and was completed by interviewing the patients and their caregivers. A GDS score of 1 indicates no cognitive impairment; 2 indicates a very mild cognitive impairment, 3 as mild, 4 as moderate, 5 as severe, 6 as very severe, and 7 as worst impairment.

The 24-item Hamilton Rating Scale for Depression (HRSD-24) was utilized to assess patient depressive symptoms [34]. In the HRSD-24 version, a total score of 8 or less was classified as no depression, 9–20 as suspicious depression, 21–35 as moderate or mild depression, and above 35 as severe depression.

The Hamilton Anxiety Scale (HAMA) was used to evaluate patient anxiety, and there were 14 items on this scale [35]. The scale adopts a 5-point scoring method ranging from 0 to 4 points. A total score less than 7 indicated no anxiety, 7–13 was classified as possible anxiety, 14–20 was anxiety, 21–28 was classified as significant anxiety, above 29 was classified as severe anxiety.

### 2.2. Measurement of Blood Levels of Amyloid Peptides and Tau-pT181 Proteins

Amyloid-β and Tau-pT181 levels in blood specimens were measured using the enzyme-linked immunosorbent assay (ELISA) methods. The ELISA kits for Aβ_40_ (KHB3481), Aβ_42_ (KHB3441), and Tau-pT181 proteins (KHO0631) were obtained from Invitrogen (Carlsbad, CA, USA). All blood specimens were collected between 6:00 and 9:00 a.m. in a fasted state. Heparin anticoagulant blood was collected by a vacuum tube and centrifuged at 3000 rpm for 10 min for plasma separation. The samples were then aliquoted and stored at −80 °C. ELISA assays were conducted within one week at three repeats for each specimen.

### 2.3. The Intervention of Sleep Disorders and Dementia

All patients enrolled in this study received a standard anti-dementia medicine donepezil (5–10 mg, QN). Patients with sleep-disordered breathing were managed by continuous positive pressure ventilation.

### 2.4. Data Collection and Statistical Analysis

Data of biometrics, behavioral and neuropsychological assessments, and Amyloid-β and Tau-pT181 tests were obtained at the enrollment before treatment and at the end of the 6-month treatment. Statistical analysis was performed using SPSS software (version 28.0, Chicago, IL, USA) and the figure graphs were generated using the GraphPad Prism software (version 9.0.0, San Diego, CA, USA). Statistical comparison among multiple groups was conducted using two-tail ANOVA analysis. The comparison of the parameters between pre- and post-treatment was analyzed using a paired *t*-test. Pearson correlation analysis was used to determine the correlation among all parameters. The Receiver Operator Characteristic (ROC) analysis was used to identify a prediction factor for sleep quality disorder.

## 3. Results

### 3.1. Patients Population and Clinical Parameters

**Figure 1:**
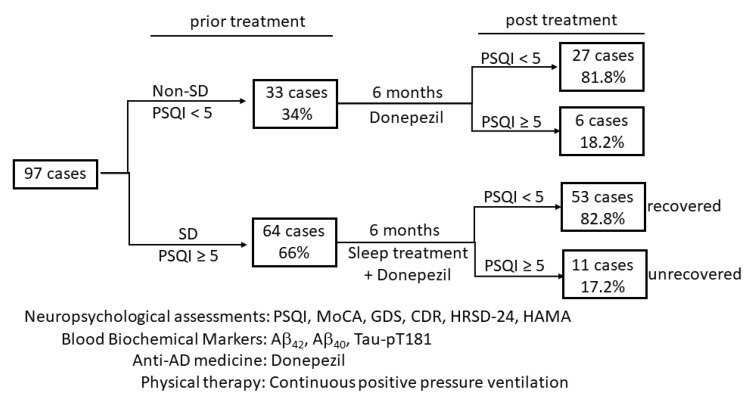
A schematic illustration of the study design and protocol. A total of 97 patients participated; 64 patients were diagnosed with sleep disorders and were subjected to sleep interventions in addition to donepezil treatment. The other 33 cases without sleep disorders received donepezil only. After a 6-month treatment, patients were re-assessed and divided into recovered or unrecovered subgroups groups based on the Pittsburgh Sleep Quality Index (PSQI) scores. SD: sleep-disorder.

A total of 97 AD-related dementia patients were enrolled in this study, of whom 33 patients without sleep-disordered breath were included as the control group, and 64 cases with sleep-disordered breathing were set as the treatment group (Figure 1). All biometric data are summarized in Table 1. All subjects had a CDR score of 1–2 (mild–moderate dementia) and a MoCA score of 12–26 (cognition impairment). Sixty-three patients (64.9%) were scored as suspicious depression (HRSD-24 score 9–20). Fifty-three (54.6%) patients were scored as anxiety (HAMA score 14–18), and 42 (43.3%) patients were scored as possible anxiety (HAMA score 7–13). Interestingly, a negative and robust correlation (Persons *r* = −0.573) was discovered between the education level and dementia onset age (Table 2), indicating that higher educated patients tended to have an early onset age of mild–moderate dementia.

### 3.2. Sleep Quality Was Associated with Neuropsychological Symptoms and Blood Biomarkers

We first assessed the entire group (97 cases) for the correlation of sleep quality using the PQSI score with the neuropsychological status and blood biomarkers. As shown in Table 2, PQSI score significantly correlated with HAMA and HRSD-24 scores, as well as the Blood Aβ_42/40_ ratio and Tau-pT181 level (Table 2). Further analysis revealed a significant correlation among Tau-pT181 level, Aβ_42/40_ ratio, HAMA, and HRSD-24 scores. These data indicate that sleep quality is associated with depression and anxiety behavior, as well as blood biomarkers in MCI patients due to Alzheimer’s disease.

**Figure 2:**
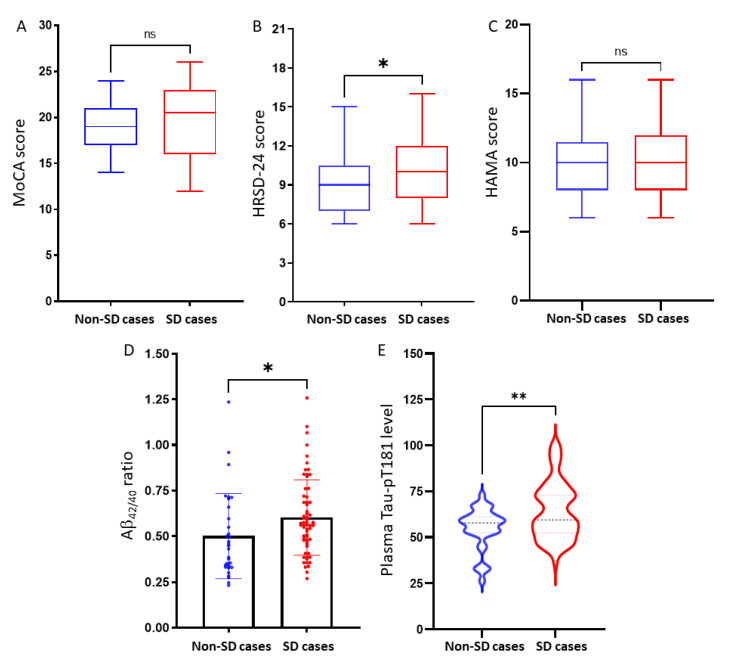
The differences of neuropsychological scores and blood biomarkers between SD cases and non-SD cases, including MOCA score (**A**), HRSD-24 score (**B**), HAMA score (**C**), Aβ_42/40_ ratio (**D**), and Tau-pT181 level (**E**). The asterisk indicates a significant difference between the groups (* *p* < 0.05, ** *p* < 0.01, Student *t*-test).

We then compared neuropsychological scores and blood biomarkers between patients with or without sleep-disordered breathing conditions (non-SD vs. SD cases). As shown in Figure 2, HRSD-24 score, Aβ_42/40_ ratio, and Tau-pT181 levels were significantly higher in patients with sleep-disordered breathing (SD cases) than those in non-SD cases; however, MoCA and HAMA scores did not show any statistically significant differences. These data demonstrated that HRSD-24 score, blood Aβ_42/40_ ratio, and Tau-pT181 level were strongly associated with sleep-disordered breathing in MCI patients due to Alzheimer’s disease.

### 3.3. Sleep Treatment Improves COGNITION and Relieves Anxiety

Patients with sleep-disordered breathing (64 cases) were treated with continuous positive pressure ventilation for six months in addition to the standard care of anti-Alzheimer’s disease medicine donepezil. Pearson correlation analysis revealed a strong correlation among the PSQI, neuropsychological scores, blood Aβ_42/40_ ratio, and Tau-pT181 levels (Table 3), similar to the correlations for the entire cohort (Table 2).

**Figure 3:**
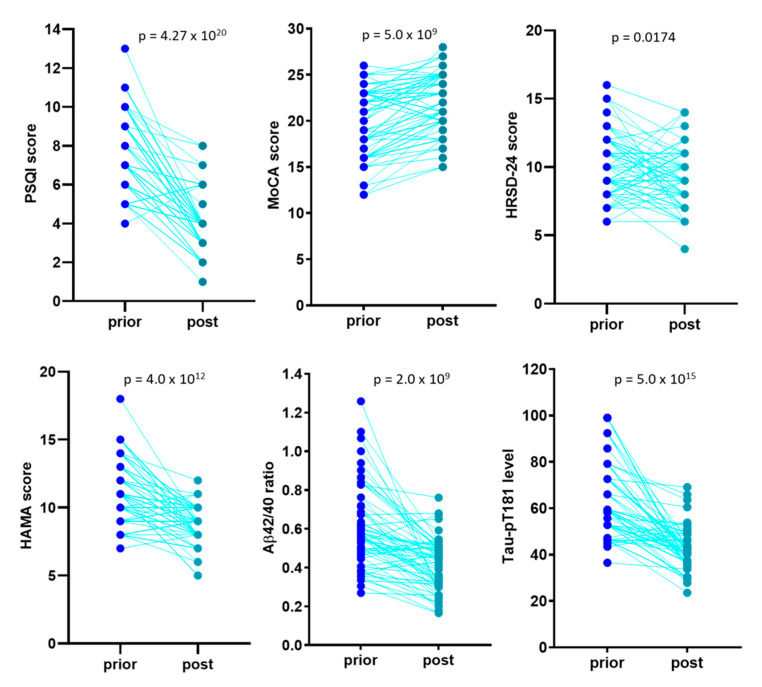
Sleep treatment improves neuropsychological scores, blood Aβ_42/40_ ratio, and Tau-pT181 protein level. Data were presented as case-controlled pairs of prior- and post-treatment values. The *p* values were derived from a pair-wise *t*-test.

**Figure 4:**
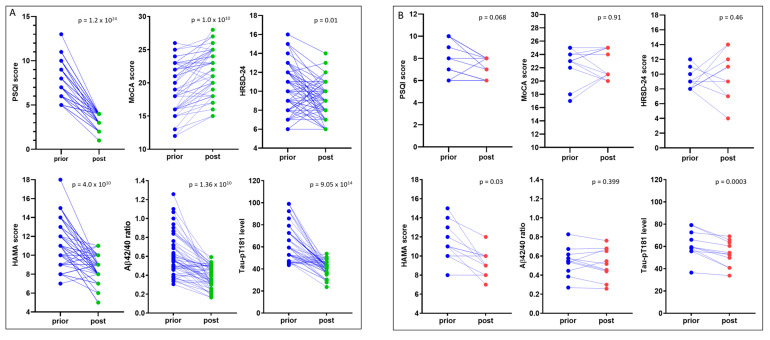
Different improvements in recovered (panel **A**) and unrecovered (panel **B**) subgroups. The neuropsychological scores, blood Aβ_42/40_ ratio, and Tau-pT181 protein level were presented as case-controlled pairs of prior- and post-treatment values. The *p* values were derived from a pair-wise t-test.

After a 6-month sleep intervention, a significant improvement was achieved for all the neuropsychological scores, blood Aβ_42/40_ ratio, and Tau-pT181 levels (Figure 3). Among these patients, 53 cases (82.8%) showed a complete recovery in PSQI score (<5) but 10 cases were only showed a slight improvement (PSQI > 5 post-treatment). One patient had a PSQI increased from prior to treatment of 6 to post-treatment of 8. After separating these two subgroups, a substantial improvement was observed for the recovered cases in MoCA, HRSD-24, and HAMA scores after sleep treatment. blood Aβ_42/40_ ratio and Tau-pT181 levels were also significantly reduced after the 6-month sleep treatment in this subgroup (Figure 4A). In the unrecovered subgroup, although HAMA scores and blood Tau-pT181 levels reduced considerably, MoCA and HRSD-24 scores, as well as blood Aβ_42/40_ ratio, did not improve significantly (Figure 4B).

**Figure 5:**
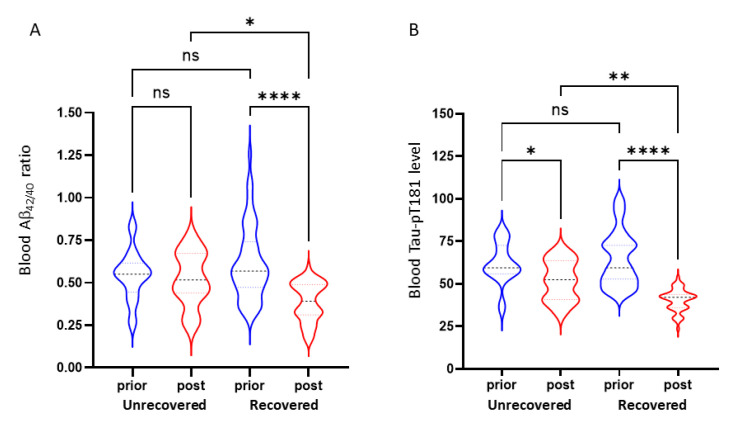
Blood Aβ_42/40_ ratio (panel **A**) and Tau-pT181 protein levels (panel **B**) were reduced in recovered subgroups. Group comparison was conducted using Student *t*-test, and the asterisks indicate a significant difference between the subgroups as indicated. * *p* < 0.05; ** *p* < 0.01; **** *p* < 0.0001; ns, not significant.

We then compared blood Aβ_42/40_ ratio and Tau-pT181 protein levels prior- or post-sleep treatment in unrecovered and recovered subgroups. As shown in Figure 5A, the Aβ_42/40_ ratio showed a significant reduction in the recovered subgroup but not in the unrecovered subgroup. In contrast, Tau-pT181 protein levels showed a significant reduction in both recovered and unrecovered subgroups (Figure 5B). However, the recovered subgroups showed a much lower Aβ_42/40_ ratio and Tau-pT181 protein than the unrecovered subgroups. These data suggest that a full recovery of sleep quality is necessary to reduce the blood Aβ_42/40_ ratio and improve recognition and depression status.

### 3.4. Blood Amyloid-β_42/40_ Ratio Is a Predictive Factor for Sleep Quality in MCI Patients

**Figure 6:**
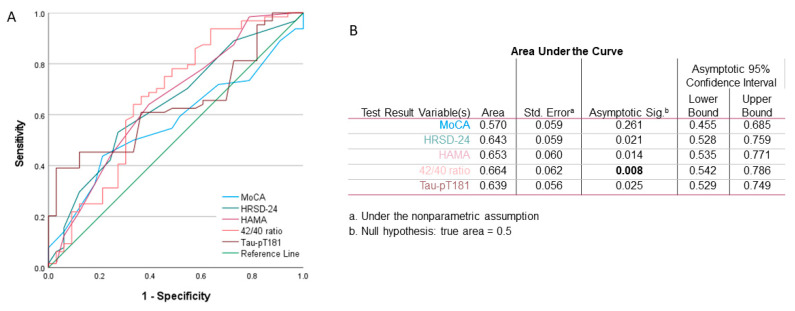
Receiver Operator Characteristic (ROC) analysis indicates that the Aβ_42/40_ ratio has the highest potential for predicting sleep quality. (**A**) The ROC curves for the variables of Aβ_42/40_ ratio, Tau-pT181, MoCA, HRSD-24, and HAMA scores. (**B**) Summary of the statistical values derived from the ROC curve analysis.

Finally, we determined if the neuropsychological score, blood Aβ_42/40_ ratio, and Tau-pT181 protein level had any predictive value as a risk factor for sleep quality. The ROC analysis included the prior-treatment values from all 97 patients. Sleep quality was set as the dependent variate (PSQI < 5). As shown in Figure 6, Aβ_42/40_ ratio had the highest significance as a predicting factor over HAMA and HRSD-24 scores and Tau-pT181 protein level. These data suggest that Aβ_42/40_ ratio is an important factor associated with sleep disorder in MCI patients due to Alzheimer’s disease.

## 4. Discussion

In this study, our main findings are listed below: (1) a very strong and negative correlation was discovered between education level and MCI onset age; (2) a strong correlation was identified among sleep quality (PSQI score), depression (HRSD-24 score), anxiety (HAMA score), blood Aβ_42/40_ ratio and Tau-pT181 levels; (3) patients with sleep disorder-breathing exhibited a worse HRSD-24 score and higher levels of blood Aβ_42/40_ ratio and Tau-pT181 levels; (4) sleep treatment for sleep-disordered breathing improved all neuropsychological scores, Aβ_42/40_ ratio, and Tau-pT181 levels; (5) a complete recovery of sleep quality is critical to fully improve the MoCA and HRSD-24 score and to reduce the Aβ_42/40_ ratio; and (6) blood Aβ_42/40_ ratio showed the highest significance in predicting sleep quality. These data suggest that sleep treatment improved neuropsychological symptoms and reduced blood Aβ_42/40_ ratio and Tau-pT181 protein levels in MCI patients due to Alzheimer’s disease.

Accumulating evidence has demonstrated sleep disordered-breathing as a clinical contributing factor in patients with MCI during Alzheimer’s disease development and progression [13,15]. However, very few studies reported the correlation of sleep quality (PSQI score) with neuropsychological scores that occur at any stage in Alzheimer’s disease [36]. This study assessed patient sleep quality with the PSQI and neuropsychological scores. Our results revealed that PSQI scores correlated significantly with HRSD-24 and HAMA scores, which were supported by a cross-sectional study showing an inverse correlation between sleep length and anxiety symptoms in Alzheimer’s disease patients [37] and by a recent study showing a close correlation between PSQI scores and depression in Alzheimer’s disease patients [38].

So far, there is a paucity of literature about the correlation of sleep quality (PSQI scores) with MoCA, GDS, and CDR scores. However, a linear correlation (coefficient of multiple correlations at 0.307–0.34) between sleep quality and GDS score was reported in Alzheimer’s disease patients with moderate to severe dementia (CDR 2–3) [39], indicating a possible connection of sleep disturbance with global deterioration scale at a late stage of AD patients. Also, lower MoCA scores were found in patients with obstructive sleep apnea-hypopnea syndrome (OSAHS) than non-insomnia patients [40], indicating a potential effect of sleep disturbance on cognitive impairment in patients without Alzheimer’s disease. Meanwhile, our results showed that treating SDB patients with continuous positive pressure ventilation significantly improved the MoCA, HRSD-24 and HAMA scores. Significantly, our results are supported by a previous report derived from a randomized clinical trial in patients with Alzheimer’s disease [22]. Therefore, sleep improvement might be able to slow down the process of Alzheimer’s disease (15), although further investigation is warranted to determine the clinical significance of sleep disordered-breathing in different phases of Alzheimer’s disease.

Aberrant aggregation of phosphorylated Tau protein is one of the major pathogenic factors in the development and progression of Alzheimer’s disease [3,8]. Also, excessive shedding and deposition of amyloid peptides have been considered a critical factor in Alzheimer’s disease [3,14]. Studies in mouse models of Alzheimer’s disease and humans have convincingly demonstrated a tight association between sleep disorders and Alzheimer’s disease in terms of Tau proteins [14,41]. Increased cerebrospinal fluid and blood levels of Amyloid-β peptide and phosphorylated Tau protein was reported in patients with sleep disturbances, including SDB [15], and have been considered biomarkers for disease progression [42,43,44]. A recent meta-analysis indicated that blood levels of Aβ_42/40_ ratio and Tau-pT181 protein strongly predicted the Aβ-PET status in patients [45] and that blood Aβ_42/40_ ratio was even recommended as a cost-effective marker for early AD pathological screening [46]. Our data also showed a strong correlation between PSQI scores and blood Aβ_42/40_ ratio and Tau-pT181 levels before and after sleep intervention in this study. Interestingly, we also found that Aβ_42/40_ ratio and Tau-pT181 levels correlated with HRSD-24 and HAMA scores. Although it is hard to postulate their causative relationship among these correlations, they are the first clue for further investigation to determine the correlation of Aβ_42/40_ ratio and Tau-pT181 level with cognition/dementia or depression/anxiety during Alzheimer’s disease progression.

For Alzheimer’s disease, a fundamental challenge is determining if sleep intervention improves neuropsychological status and reduces the Tau protein burden in patients [24]. Our data demonstrated that sleep intervention resulted in a significant reduction of blood Tau-pT181 levels. More specifically, patients with or without a full recovery achieved a substantial decrease in Tau-pT181 levels. In contrast to the Tau-pT181 decline, Aβ_42/40_ ratio was not significantly reduced in unrecovered patients, indicating that a complete recovery is critical in lowering the Aβ_42/40_ ratio.

Our data are supported by other clinical studies showing a 50% increase of Tau-pT181 proteins in human cerebrospinal fluid after sleep deprivation [18]. These results indicate a potential causative relationship between sleep disturbance and accumulation of Tau-pT181 in Alzheimer’s disease. Aberrant accumulation of Tau protein occurs due to an imbalanced production and clearance process reported recently [14,24]. A high-quality sleep-wake cycle is vital for successfully clearing these pathogenic molecules [21,23,47]. Therefore, it is plausible that sleep intervention at the preclinical stage of Alzheimer’s disease has a strong potential to prevent or slow down disease progression [48].

There were a few limitations in our study. First, we utilized the most economical but reliable blood biomarkers, Aβ_42/40_ ratio and Tau-pT181 level [42,45,46]. However, more precise and accurate methods are available in the field, such as HPLC measurement in CSF or blood specimens [49] and Florbetapir (^18^F)-positron emission tomography (PET) [50]. With advanced imaging technologies like functional MRI and ^18^F (or ^11^C-PIB)-PET, more precise brain tissue changes after sleep treatment will be clearly understood for mechanistic analysis. Second, we used the subjective assessments of sleep quality with PSQI and neuropsychological evaluation system of MoCA, GDS, CDR, HRSD-24, HAMA, which have been widely used in the field as standard tools in clinical practice. Although the self-assessment system by older patients might not reflect their actual sleep quality compared to a subjective measurement like polysomnography [51], the alterations of PSQI scores between prior- and post-treatment were analyzed using a paired *t*-test (longitudinal self-comparison) so that the derivations among individuals were minimized. It is plausible that objective measurements with advanced technology for sleep monitoring will significantly strengthen our conclusion. Third, 11 cases did not fully recover after a 6-month treatment. We are still monitoring these cases for a more extended intervention and follow-up until a possible complete recovery within two years.

## 5. Conclusions

This is a longitudinal study with 97 cases of mild–moderate cognitive impairment patients due to Alzheimer’s disease. Of these, 64 subjects were treated due to sleep-disordered breathing. The sleep quality scores were significantly correlated with depression and anxiety scores, blood Aβ_42/40_ ratio, and Tau-pT181 protein levels. Sleep treatment significantly improved sleep quality and enhanced cognition, relieving anxiety, and reduced blood Aβ_42/40_ ratio and Tau-pT181 protein levels. A full recovery of sleep quality was critical to improving cognition and depression status. Our data provided valuable evidence that sleep intervention might be feasible to improve neuropsychological symptoms and reduce disease progression when implicated in the early phase of Alzheimer’s disease.

## Figures and Tables

**Table 1:** Patient Biometrics Parameters.

	All Cases			SD Cases with Treatment		
	Non-SD Cases	SD Cases	*p*-Value	Un-Recovered	Recovered	*p*-Value
Case number (%)	33 (34%)	64 (66%)		11 (17.2%)	53 (82.8%)	
Age (year)	73 (66–81)	73 (63–81)	n.s.	73 (67–78)	73 (63–81)	n.s.
Onset age (year)	69 (63–79)	71 (61–79)	n.s.	69 (63–79)	71 (61–79)	n.s.
Sex (Male/Female)	M18/F15	M30/F34	n.s.	M6/F5	M24/F29	n.s.
Disease length (month)	32 (19–45)	32 (16–59)	n.s.	32 (19–45)	32 (16–59)	n.s.
Secondary education	4 (2–11)	4 (0–11)	n.s.	4 (2–11)	4 (0–11)	n.s.
Body Mass Index	23.7 (16.7–29.2)	23.6 (15.48–33.02)	n.s.	23.7 (16.7–29.2)	24.5 (15.48–33.02)	n.s.

Note: The values for clinical assessments were shown as the Median (range). The *p*-value was derived from Student *t*-test analysis between non-recovered and recovered groups except Χ^2^ test for sex. n.s., not significant. SD, sleep disorder.

**Table 2:** Pearson Correlation Coefficient.

Correlation Pair	Pearson *r*	*p*-Value
Education vs. Onset age	−0.573	8.53 × 10^−10^
PSQI vs. HAMA	0.467	1.42 × 10^−6^
PSQI vs. HRSD-24	0.353	0.0004
PSQI vs. Aβ_42/40_ ratio	0.348	0.0005
PSQI vs. Tau-pT181	0.424	1.52 × 10^−5^
HRSD-24 vs. HAMA	0.419	1.93 × 10^−5^
HRSD-24 vs. Aβ_42/40_ ratio	0.506	1.24 × 10^−7^
HRSD-24 vs. Tau-pT181	0.643	1.30 × 10^−12^
HAMA vs. Aβ_42/40_ ratio	0.506	1.28 × 10^−7^
HAMA vs. Tau-pT181	0.555	3.61 × 10^−9^
Aβ_42/40_ ratio vs. Tau-pT181	0.588	2.34 × 10^−10^

Note: 24-h Hamilton Rating Scale for Depression: HRSD-24 and Hamil-ton Anxiety Rating Scale: HAMA, Amyloid β peptide-42/40: Aβ_42/40_.

**Table 3:** Pearson Correlation Coefficient.

Correlation Pair	Pearson *r*	*p*-Value
Education vs. Onset age	−0.709	5.53 × 10^−11^
PSQI vs. HAMA	0.488	4.22 × 10^−4^
PSQI vs. HRSD-24	0.268	0.032
PSQI vs. Aβ_42/40_ ratio	0.311	0.012
PSQI vs. Tau-pT181	0.328	0.008
MoCa vs. GDS	−0.409	0.0008
GDS vs. Aβ_42/40_ ratio	−0.316	0.011
HRSD-24 vs. HAMA	0.407	0.0008
HRSD-24 vs. Aβ_42/40_ ratio	0.580	4.96 × 10^−7^
HRSD-24 vs. Tau-pT181	0.709	5.13 × 10^−11^
HAMA vs. Aβ_42/40_ ratio	0.526	7.98 × 10^−6^
HAMA vs. Tau-pT181	0.663	2.43 × 10^−9^
Aβ_42/40_ ratio vs. Tau-pT181	0.772	7.84 × 10^−14^

Note: Montreal Cognitive Assessment: MoCA, Geriatric Depression Scale: GDS, Clinical De-mentia Rating: CDR.
